# Use of Botulinum Toxin A to Treat Chemotherapy-Induced Raynaud’s Phenomenon

**DOI:** 10.7759/cureus.12511

**Published:** 2021-01-05

**Authors:** Thrisha K Potluri, Frank G Lee, Ethan Song, Sean J Wallace, Nathan Miller

**Affiliations:** 1 Department of Plastic Surgery and Reconstructive Medicine, University of South Florida Morsani College of Medicine, Tampa, USA; 2 Division of Plastic & Reconstructive Surgery, Lehigh Valley Health Network, Allentown, USA

**Keywords:** raynaud disease, chemotherapy, botulinum toxin type a

## Abstract

Raynaud’s phenomenon (RP) is a vasospastic disorder of the digital blood vessels leading to pain, paresthesias, and pallor in response to cold or stress. RP can develop secondary to a number of pathologies or factors, including the use of chemotherapy agents. Typical first-line therapies for secondary RP may be contraindicated in patients with certain comorbidities. Here, we discuss a case in which botulinum toxin A (BTX-A) was used to treat chemotherapy-induced RP in a patient with non-small cell lung cancer (NSCLC). We provide a review of the existing literature on the clinical course and treatment modalities, including the use of BTX-A, for patients with secondary RP.

A 56-year-old female with NSCLC received treatment with bevacizumab and pemetrexed. Her initial symptoms included progressive discoloration and pain in her fingertips, which hastily progressed to ischemia and subsequent dry gangrene. She was diagnosed with chemotherapy-induced RP, but traditional management options were complicated by acute congestive heart failure. BTX-A injections were administered at key locations on the wrist and hand, significantly improving her symptoms and slowing the progression of the gangrenous changes.

RP can develop as sequelae of chemotherapy regimens. Clinical management may be complicated by underlying pathology and/or patient symptoms. BTX-A injections are an excellent non-operative therapeutic option for patients with secondary RP in cases where mainstay therapies may be contraindicated, thus decreasing pain, improving patient quality of life, and slowing the progression of gangrenous changes.

## Introduction

Raynaud’s phenomenon (RP) is a well-characterized vasospastic condition in which patients experience transient ischemia of distal circulation. The constellation of clinical signs and symptoms includes pain, pallor, paresthesias, digital ulceration, and may even lead to gangrene [1-3]. While primary RP is idiopathic, there have been many noted etiologies of secondary RP syndrome, including combined chemotherapeutic regimens and paraneoplastic-associated malignancies [2, 4-6].

Management of chemotherapy-induced RP begins conservatively with behavioral changes. Once the symptoms progress, medical therapies are indicated and traditional modalities include the use of calcium channel blockers (CCBs), PDE-5 (phosphodiesterase type 5) inhibitors, nitrates, and prostacyclins [1, 3-4]. For severe or refractory cases of RP, more invasive therapies including nerve blocks, digital sympathectomies, and/or ultimately amputations have been described [1-3, 7]. Conventional treatments of secondary RP have shown mixed results and are often limited by efficacy, side effects, and contraindications to chronic medical comorbidities [1, 8].

Injection of botulinum toxin A (BTX-A) as a treatment option for RP has gained increasing prominence as an effective, viable therapy [3, 9-10]. Recent investigations have shown improvement in pain control, numbness, blood flow, and ulcer healing. In this report, we present the usage of BTX-A at key locations on the wrist and volar hand as symptomatic treatment for chemotherapy-induced RP in a 56-year-old woman with stage III non-small cell lung cancer (NSCLC) who developed heart failure and RP following induction of her chemotherapy regimen.

## Case presentation

A 56-year-old female with NSCLC undergoing medical oncologic therapy with bevacizumab and pemetrexed presented to the emergency room with a one-month history of progressively worsening blue discoloration and pain in her distal fingertips and toes. She had recently completed her 21st cycle of bevacizumab/pemetrexed. She had never used tobacco products and had no significant pre-existing medical conditions other than osteopenia. Physical examination revealed cool, dusky discoloration of the fingertips and toes bilaterally and prolonged capillary refill despite intact pulses. Consultation with Vascular Surgery and Rheumatology confirmed a diagnosis of chemotherapy-induced RP. Angiography of the bilateral upper extremities revealed no major arterial stenosis; however, distal perfusion of fingertips was poor. Administration of nitroglycerin during angiography provided no significant improvement in perfusion distally. First-line medical therapies were initiated, which included the commencement of amlodipine and enoxaparin. Plastic, Reconstructive, and Hand Surgery (PRS) was not consulted during this hospitalization.

Two weeks later, she returned to the emergency department with shortness of breath secondary to decompensated congestive heart failure (CHF) found to be a result of her chemotherapy. Her digital symptoms had worsened. An examination found her to have started the development of dry gangrene involving the distal phalanges of multiple digits bilaterally. Secondary to chemotherapy-induced decompensated CHF, traditional medical therapies to improve digital perfusion including sildenafil, CCBs, nitrates, and beta-blockers were contraindicated. Anticoagulants were continued and included enoxaparin and the addition of clopidogrel.

**Figure 1:**
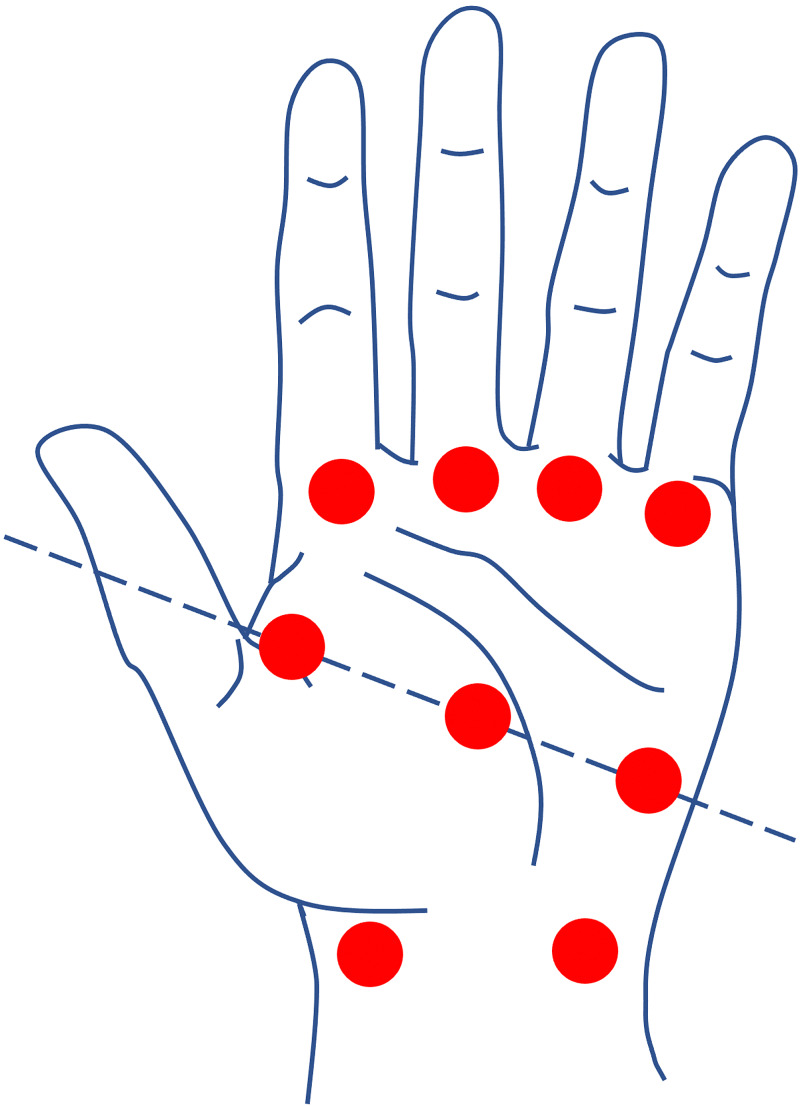
Schematic representing botulinum toxin A injection sites. The Dashed line represents Kaplan’s cardinal line.

**Figure 2:**
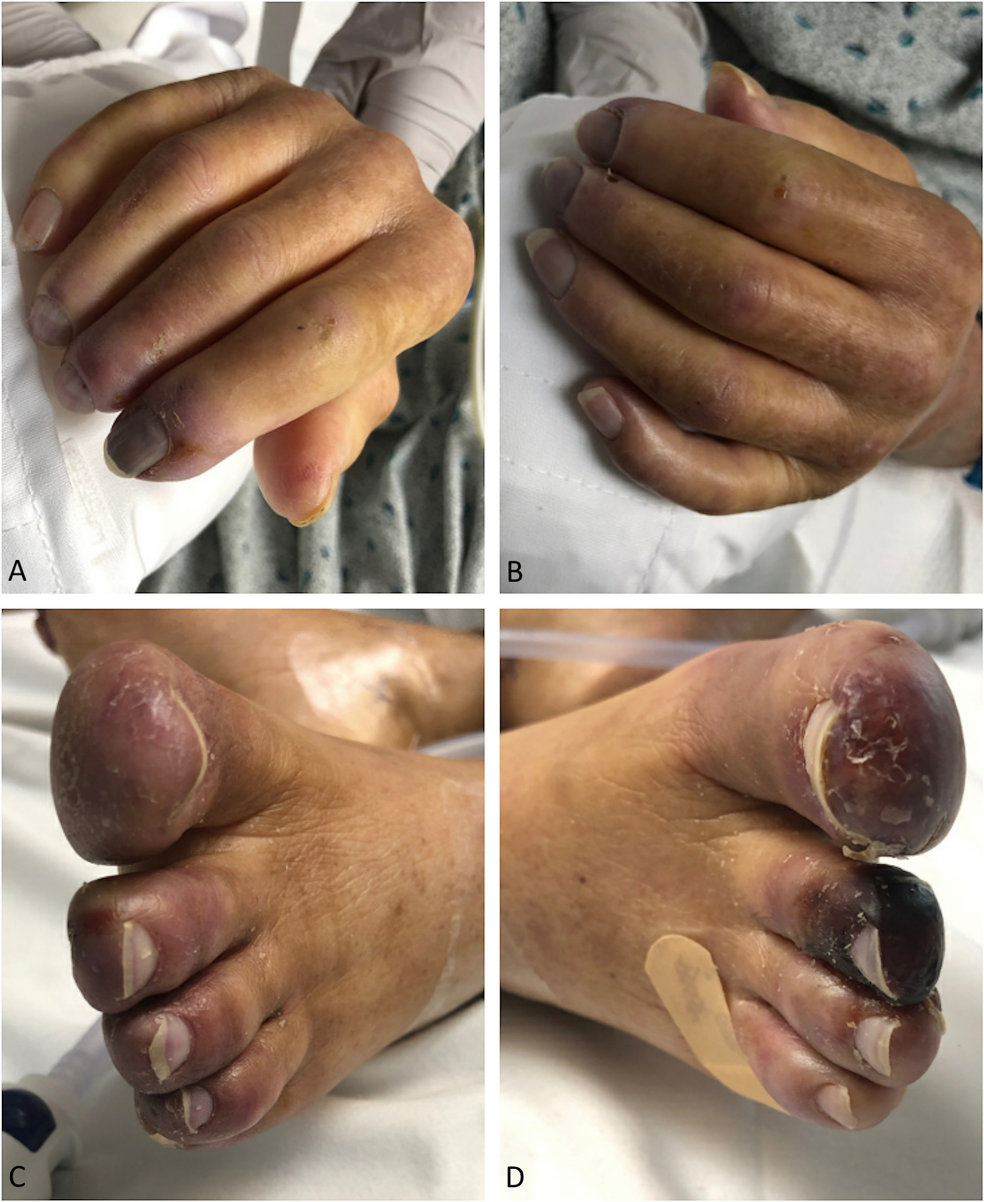
A-B. Clinical photograph of hands and digits one year after botulinum toxin A treatment of chemotherapy-induced RP. C-D. Clinical photograph of feet taken at one year, treated only with first-line medical therapies including enoxaparin, sildenafil, nitrates, nifedipine, and betadine.

PRS was consulted for further assistance with management and wound care recommendations. Administration of BTX-A was proposed and approved by the patient’s insurance. A total of 90 units per wrist and hand were injected intradermally equally over nine locations, with 10 units per site (Figure 1). Preparation of the BTX-A included titration of 100 units in 4 ml of preservative-free 0.9% sterile normal saline solution, giving a dilution of 25 units per 1 ml (or 2.5 units per 0.1 ml). These included superficial to radial artery at the proximal wrist crease, superficial to the ulnar artery at the proximal wrist crease, three sites along Kaplan’s cardinal line, and at the A1 pulleys located at the base of the index, long, ring, and small fingers. Over the next five-to-seven days, the patient reported subjective improvement in symptoms, most notably severe pain, and objectively, visible improvement in temperature and color of the digits. There was no progression of gangrene of her fingertips noted on follow-up visits in the clinic, but the disease continued to progress in her toes (Figure 2). Local wound care was provided and assured with regularly scheduled outpatient visits. While the BTX-A was functioning, she reported high satisfaction with the relief from pain and improvement in ease of activities of daily living with follow-up to 18 months. Unfortunately, the patient went on to succumb to her NSCLC.

## Discussion

RP is a common disorder with episodic digital vasospasm following cold exposure or stress, leading to pain, paresthesias, and cyanosis. While primary RP is idiopathic, many factors are associated with secondary RP syndrome, including combined chemotherapeutic agents. The specific pathophysiology of chemotherapy-induced RP is still not fully understood, although numerous studies have proposed the phenomenon is related to the vasospasm of small vessels [6, 11-12]. Some evidence suggests chemotherapy may induce vascular toxicity and endothelial dysfunction leading to secondary RP [13]. Other studies posit drug toxicity leads to abnormal and prolonged vasoconstrictor response, impaired non-neurogenic autoregulation, and peripheral neuropathy [4-5, 12-13]. Various chemotherapeutic agents have been associated with secondary RP, including cisplatin, vincristine, and bleomycin-based regimens. [4-5]. In this instance, bevacizumab was the likely offending agent.

Patients with secondary RP often present with more severe disease and pose a greater challenge in clinical management [1, 3, 7]. Symptoms and other manifestations of underlying pathology in secondary RP may exacerbate digital vasospasm and impact treatment modality, as evidenced in this report. Traditional treatment modalities include beginning conservatively before progressing to pharmacologic management with CCBs, PDE-5 inhibitors, topical nitrates, prostacyclin, iloprost, and/or anticoagulants [1-3]. These mainstay treatments do have limitations and may be contraindicated in patients with certain comorbidities. In this report, the patient was primarily hospitalized for an acute CHF exacerbation. Due to the decompensated CHF, alternative management needed to be considered, which included the utilization of BTX-A to improve localized perfusion to the hands and digits.

The mechanism of action by which BTX-A exerts its therapeutic effects in RP patients is not yet completely understood; however, it is well-described that BTX-A inhibits acetylcholine release, leading to inhibition of neurotransmitter-induced vasospasms, reduced vasoconstriction and pain, and relief of symptoms in vascular conditions [3, 8, 14]. Other proposed mechanisms of action include increased perfusion within myocytes [8], inhibition of alpha-adrenergic receptors in vascular smooth muscle in cold conditions [15], and/or inhibited release of downstream pain mediators like glutamate, calcitonin-gene related peptide, and substance P [10]. The first case series to describe BTX-A for RP showed improvement in pain and perfusion in patients with primary and secondary RP [9]. Other studies have reported ulcer healing in RP patients with acute digital ischemia, as well as reduced discoloration, increased surface temperatures, and mean hand strength. [7, 8, 15-17]. A 2016 systematic review showed promising signs in metrics like pain and Disabilities of the Arm, Shoulder, Hand (DASH) score [18]. A case report on BTX-A hand injections to treat paraneoplastic RP secondary to lung malignancy also showed halted progression and improvement of symptoms, similar to our patient [19].

While previous data have shown a benefit of this treatment modality in improving digital perfusion [8, 10, 14-15], we provide further evidence of BTX-A as an excellent non-operative local therapeutic option for chemotherapy-induced digital RP, particularly in patients who have contraindications to established first-line treatments. We hypothesize that the BTX-A injections may have also played a protective role and prevented progression of RP and digital ischemia, as evidenced by the deterioration of the patient’s untreated toes compared to halted progression in the BTX-A treated hands.

Standardization of BTX-A injection sites in the treatment of chemotherapy-induced RP has not been well-described and can be a challenging consideration for internal validity. Suggestions for appropriate workup for BTX-A injections in RP have been made, including angiography to rule out pathologies readily fixable by a vascular bypass. Noted contraindications to BTX-A injections include allergy, pregnancy, infections, drugs that alter neuromuscular transmission, and pathologies like myasthenia gravis and Lambert-Eaton [20]. While further research is necessary to determine the mechanism of action, dosage and frequency, injection sites, and efficacy relative to pharmacologic or surgical treatments, BTX-A is an excellent treatment modality and adjunct for RP secondary to chemotherapy. Additional considerations include cost and coverage by insurance, as its application is being approved more with increasing evidence supporting outcomes of BTX-A therapy for complicated RP. We also recommend utilizing a visual analog scale (VAS) or another similar metric to standardize satisfaction level and compare pre- and post-injection. In conclusion, BTX-A injections can extend to additional use cases in the setting of chemotherapy-induced RP and may have preventative and therapeutic potential when other treatment modalities of RP may be inappropriate.

## Conclusions

We report a case of chemotherapy-induced Raynaud’s phenomenon treated with local BTX-A in an adult female with a history of NSCLC. She underwent several cycles of bevacizumab therapy, which contributed to her secondary RP. She was treated with administration of BTX-A and ultimately discharged with improved functional and aesthetic outcomes through 18 months of follow-up. BTX-A is a promising nonoperative treatment modality and/or adjunct for patients who have contraindications to calcium channel blockers, PDE-5 inhibitors, and nitrates.

## References

[1] Stringer T, Femia AN (2018). Raynaud's phenomenon: current concepts. Clin Dermatol.

[2] Block JA, Sequeira W (2001). Raynaud's phenomenon. Lancet.

[3] Herrick AL, Wigley FM (2020). Raynaud's phenomenon. Best Pract Res Clin Rheumatol.

[4] Khouri C, Blaise S, Carpentier P, Villier C, Cracowski JL, Roustit M (2016). Drug-induced Raynaud's phenomenon: beyond β-adrenoceptor blockers. Br J Clin Pharmacol.

[5] Vogelzang NJ, Bosl GJ, Johnson K, Kennedy BJ (1981). Raynaud's phenomenon: a common toxicity after combination chemotherapy for testicular cancer. Ann Intern Med.

[6] Marie I, Levesque H, Plissonnier D, Balguerie X, Cailleux N, Courtois H (2000). Digital necrosis related to cisplatin in systemic sclerosis. Br J Dermatol.

[7] Dhaliwal K, Griffin MF, Salinas S, Howell K, Denton CP, Butler PEM (2019). Optimisation of botulinum toxin type a treatment for the management of Raynaud's phenomenon using a dorsal approach: a prospective case series. Clin Rheumatol.

[8] Fregene A, Ditmars D, Siddiqui A (2009). Botulinum toxin type A: a treatment option for digital ischemia in patients with Raynaud's phenomenon. J Hand Surg Am.

[9] Sycha T, Graninger M, Auff E, Schnider P (2004). Botulinum toxin in the treatment of Raynaud's phenomenon: a pilot study. Eur J Clin Invest.

[10] Neumeister MW The role of botulinum toxin in vasospastic disorders of the hand. Hand Clin.

[11] Ting JC, Fukshansky M, Burton AW (2007). Treatment of refractory ischemic pain from chemotherapy-induced Raynaud's syndrome with spinal cord stimulation. Pain Pract.

[12] Hansen SW, Olsen N, Rossing N, Rørth M (1990). Vascular toxicity and the mechanism underlying Raynaud's phenomenon in patients treated with cisplatin, vinblastine and bleomycin. Ann Oncol.

[13] McGrath SE, Webb A, Walker-Bone K (2013). Bleomycin-induced Raynaud's phenomenon after single-dose exposure: risk factors and treatment with intravenous iloprost infusion. J Clin Oncol.

[14] Motegi SI, Uehara A, Yamada K (2017). Efficacy of botulinum toxin B Injection for Raynaud's phenomenon and digital ulcers in patients with systemic sclerosis. Acta Derm Venereol.

[15] Van Beek AL, Lim PK, Gear AJ, Pritzker MR (2007). Management of vasospastic disorders with botulinum toxin A. Plast Reconstr Surg.

[16] Jenkins SN, Neyman KM, Veledar E, Chen SC (2013). A pilot study evaluating the efficacy of botulinum toxin A in the treatment of Raynaud phenomenon. J Am Acad Dermatol.

[17] Iorio ML, Masden DL, Higgins JP (2012). Botulinum toxin A treatment of Raynaud's phenomenon: a review. Semin Arthritis Rheum.

[18] Żebryk P, Puszczewicz MJ (2016). Botulinum toxin A in the treatment of Raynaud’s phenomenon: a systematic review. Arch Med Sci.

[19] Wang L, Lei Q-S, Liu Y-Y, Song G-J, Song C-L (2016). A case report of the beneficial effects of botulinum toxin type A on Raynaud phenomenon in a patient with lung cancer. Medicine (Baltimore).

[20] Gallegos JE, Inglesby DC, Young ZT, Herrera FA (2020). Botulinum toxin for the treatment of intractable Raynaud phenomenon. J Hand Surg Am.

